# Comparative evaluation of the treatment efficacy of suberoylanilide hydroxamic acid (SAHA) and paclitaxel in ovarian cancer cell lines and primary ovarian cancer cells from patients

**DOI:** 10.1186/1471-2407-6-183

**Published:** 2006-07-11

**Authors:** Jürgen Sonnemann, Jennifer Gänge, Sabine Pilz, Christine Stötzer, Ralf Ohlinger, Antje Belau, Gerd Lorenz, James F Beck

**Affiliations:** 1 Research Center of Pharmacology and Experimental Therapeutics, Ernst Moritz Arndt University, Friedrich-Loeffler-Str. 23d, D-17487 Greifswald, Germany; 2 Department of Paediatric Oncology/Haematology, Ernst Moritz Arndt University, Soldmannstr. 15, D-17487 Greifswald, Germany; 3 Department of Obstetrics and Gynaecology, Ernst Moritz Arndt University, Wollweberstr. 1, D-17475 Greifswald, Germany; 4 Department of Pathology, Ernst Moritz Arndt University, Friedrich-Loeffler-Str. 23e, D-17487 Greifswald, Germany

## Abstract

**Background:**

In most patients with ovarian cancer, diagnosis occurs after the tumour has disseminated beyond the ovaries. In these cases, post-surgical taxane/platinum combination chemotherapy is the "gold standard". However, most of the patients experience disease relapse and eventually die due to the emergence of chemotherapy resistance. Histone deacetylase inhibitors are novel anticancer agents that hold promise to improve patient outcome.

**Methods:**

We compared a prototypic histone deacetylase inhibitor, suberoylanilide hydroxamic acid (SAHA), and paclitaxel for their treatment efficacy in ovarian cancer cell lines and in primary patient-derived ovarian cancer cells. The primary cancer cells were isolated from malignant ascites collected from five patients with stage III ovarian carcinomas. Cytotoxic activities were evaluated by Alamar Blue assay and by caspase-3 activation. The ability of SAHA to kill drug-resistant 2780AD cells was also assessed.

**Results:**

By employing the cell lines OVCAR-3, SK-OV-3, and A2780, we established SAHA at concentrations of 1 to 20 μM to be as efficient in inducing cell death as paclitaxel at concentrations of 3 to 300 nM. Consequently, we treated the patient-derived cancer cells with these doses of the drugs. All five isolates were sensitive to SAHA, with cell killing ranging from 21% to 63% after a 72-h exposure to 20 μM SAHA, while four of them were resistant to paclitaxel (i.e., <10% cell death at 300 nM paclitaxel for 72 hours). Likewise, treatment with SAHA led to an increase in caspase-3 activity in all five isolates, whereas treatment with paclitaxel had no effect on caspase-3 activity in three of them. 2780AD cells were responsive to SAHA but resistant to paclitaxel.

**Conclusion:**

These *ex vivo *findings raise the possibility that SAHA may prove effective in the treatment of paclitaxel-resistant ovarian cancer *in vivo*.

## Background

Ovarian cancer is the most lethal gynaecological neoplasm, accounting for over 6% of deaths from cancer in women [1]. The standard treatment is a combination of surgery and chemotherapy, the latter usually consisting of a taxane/platinum combination. By this regimen, initial response rates of more than 80% are achieved [2]. Unfortunately, in the vast majority of women, diagnosis occurs after the disease has already disseminated beyond the ovaries. These patients typically relapse and eventually die as the tumours become refractory to treatment. Actually, drug resistance is supposed to account for therapy failure and death in more than 90% of ovarian cancer patients with advanced-stage at diagnosis [3]. Novel treatment strategies to overcome drug resistance, thus, are urgently needed.

Drug resistance is generally associated with altered gene expression in the drug-resistant cancer cells in comparison to the drug-sensitive cancer cells. Typically, resistant tumour cells show an upregulation of antiapoptotic proteins and a downregulation of proapoptotic proteins, resulting in a ratio of anti- to proapoptotic proteins favouring survival, as well as an elevated expression of drug efflux pumps, such as P-glycoprotein (Pgp) [4]. In ovarian cancer, cDNA microarray analyses of cisplatin-resistant sublines of KF-1 cells [5] and paclitaxel-resistant sublines of SK-OV-3 cells [6] have demonstrated transcriptional changes in genes coding for such proteins. Hence, treatment strategies aiming at reversing the aberrant expression of genes involved in drug response could prove effective in the management of drug-resistant ovarian cancer.

At present, considerable attention focuses on the role of epigenetic modifications in carcinogenesis and the development of drug resistance. As such, histone hypoacetylation has recently been recognized as an important type of epigenetic alteration in human cancers [7]. Histone hypoacetylation, which is associated with condensed chromatin structure and gene silencing, is the result of the aberrant actions of histone acetyltransferases and histone deacetylases. Inhibitors of histone deacetylases (HDIs) are currently emerging as a promising new class of anticancer agents: they can reactivate gene expression and prevent proliferation, activate differentiation, and/or induce apoptosis of tumour cells [8]. In so doing, they e.g. reactivate gene expression of dormant tumour suppressor genes, such as *CDKN1A *(*p21*) [9].

The potent antitumour activity of HDIs has been observed in cell lines originating from different types of human cancers as well as in studies on mice xenograft models of human neoplasia [10]. Importantly, HDIs have been shown both *in vitro *and *in vivo *to affect cancer cells while leaving normal cells comparatively unscathed [11,12]. In addition, the clinical potential of these agents has been documented by several Phase I trials of different HDIs in patients with solid tumours or leukaemias [13-18]. With respect to ovarian cancer, several preclinical studies have demonstrated the anticancer efficacy of various HDIs in cell lines *in vitro *[19-23] and in cell lines implanted into nude mice [24,25]. However, no information is available about the potential effectiveness of HDIs in primary ovarian cancer cells from patients. Herein, we demonstrate efficient *ex vivo *activity of the HDI suberoylanilide hydroxamic acid (SAHA) in patient-derived ovarian cancer cells, which display only marginal responsiveness to paclitaxel.

## Methods

### Cell culture

OVCAR-3, SK-OV-3, A2780 and 2780AD ovarian carcinoma cells were kindly provided by Dr. J. Braunger (Altana Pharma, Konstanz, Germany). SK-OV-3 cells were maintained in McCoy's 5a. OVCAR-3, A2780, and 2780AD cells were maintained in RPMI 1640. The medium of OVCAR-3 cells was supplemented with 1.5 g/l sodium bicarbonate, 1.0 mM sodium pyruvate, and 0.01 mg/ml bovine insulin; the medium of 2780AD cells was supplemented with 2.2 μM doxorubicin (Sigma, Deisenhofen, Germany). All media were supplemented with 10% fetal calf serum, 2 mM L-glutamine, 100 units/ml penicillin G sodium, and 100 μg/ml streptomycin sulphate (media and supplements were purchased from Biochrom, Berlin, Germany). Cells were cultivated at 37°C in a humidified 5% CO_2 _incubator and routinely passaged when 90–95% confluent. Cell viability was determined by the trypan blue exclusion test. Cells were regularly inspected to be free of *Mycoplasma *with *Mycoplasma *detection reagents from Roche (Mannheim, Germany).

### Isolation of primary ovarian cancer cells from ascites

The use of patient samples was approved prior to the initiation of these studies (University of Greifswald, Research Ethics Committee), and all patients signed consent forms. Ovarian cancer cells were isolated from malignant ascites collected from patients with stage III ovarian carcinomas. Of the five patients whose cells were used in this study, four were untreated, and one had been treated with a single dose of paclitaxel/carboplatin (isolate ASC-OC-4). The four untreated patients were subsequently treated with a palitaxel/carboplatin combination according to the standard protocol for the management of ovarian cancer.

The ascitic fluid was centrifuged at 3000 rpm at room temperature for 10 minutes, and the cellular pellet was washed twice in PBS. The pellet was resuspended in Ham's F12K medium (Biochrom) supplemented with 10% fetal calf serum, 2 mM L-glutamine, 100 units/ml penicillin G sodium, and 100 μg/ml streptomycin sulphate, and plated in T75 flasks. Ascitic fluid typically contains a considerable number of blood cells; however, they do not impede cell plating. After 3–4 weeks of culture, purity of the ovarian carcinoma cells was at least 80%. All experiments described here were performed at culture passages 4–7.

### Cytotoxicity assay

The assays were performed in quadruplicate in 96-well flat-bottom microtiter plates. After a 72-h incubation with either SAHA (Alexis, Grünberg, Germany) or paclitaxel (Sigma), 1/10 volume of Alamar Blue (Biosource, Solingen, Germany) solution was added and cells incubated at 37°C for an additional 3 hours. The absorbance of the wells was measured at 560/595 nm using a Wallac Victor (Perkin Elmer, Rodgau-Jügesheim, Germany) fluorometer. Results are expressed as a percentage of relative cell numbers of control cells.

### Caspase-3 activity

Caspase-3 activity was measured 24 hours after treatment with drugs using the synthetic fluorogenic substrates Ac-DEVD-AFC (Bachem, Heidelberg, Germany). Cells were lysed in 10 mM Tris-HCl, 10 mM NaH_2_PO_4_/NaHPO_4 _(pH 7.5), 130 mM NaCl, 1% Triton-X-100, and 10 mM Na_4_P_2_O_7 _and then incubated with 20 mM Hepes (pH 7.5), 10% glycerol, 2 mM DTT and 25 μg/ml Ac-DEVD-AFC at 37°C for 2 hours. The release of trifluoromethylcoumarin (AFC) was analyzed on a Wallac Victor fluorometer using an excitation/emission wavelength of 390/510 nm. Relative caspase activities were calculated as a ratio of emission of treated cells to untreated cells.

### Cytofluorometric analysis of cell death

To determine cell death, cells were harvested after a 72-h cultivation in the presence of drugs, followed by a 5-min incubation in 2 μg/ml propidium iodide (PI) (Sigma) in PBS at 4°C in the dark. PI uptake was assessed by flow cytometry analysis on a Becton Dickinson (Heidelberg, Germany) FACSCalibur using CellQuest software. 10,000 cells were analyzed in each sample; data were gated to exclude debris. Cyclosporine A (CsA; Alexis) was applied 1 hour before treatment with SAHA or paclitaxel, respectively.

### Western blot analysis

Cell were lysed on ice for 15 minutes in 40 mM Tris-HCl (pH 7.4), 150 mM NaCl, 1% Triton X-100, 0.5% sodium deoxycholate, and 0.1% SDS supplemented with a protease inhibitor cocktail (Roche) followed by brief sonification. Protein concentration was assayed using bicinchoninic acid (Pierce, Rockford, IL, USA) according to the manufacturer's instructions. For immunoblotting, 30 μg of total cellular protein per lane were separated by standard SDS-PAGE on 15% gels and electrophoretically transferred to PVDF membranes (Millipore, Eschborn, Germany). After blocking in PBS containing 5% dry milk and 0.05% Tween 20, acetylated histone H3 was immunodetected rabbit anti-acetylated histone H3 (dilution 1:10,000; Upstate Biotechnology, Lake Placid, NY, USA) polyclonal antibodies. Peroxidase-conjugated goat anti-rabbit IgGs (dilution 1:12,500; Dianova, Hamburg, Germany) followed by enhanced chemiluminescence (Amersham Biosciences, Freiburg, Germany) were used for detection.

## Results

### Comparison of the cytotoxic activities of SAHA and paclitaxel on ovarian cancer cells

**Figure 1:**
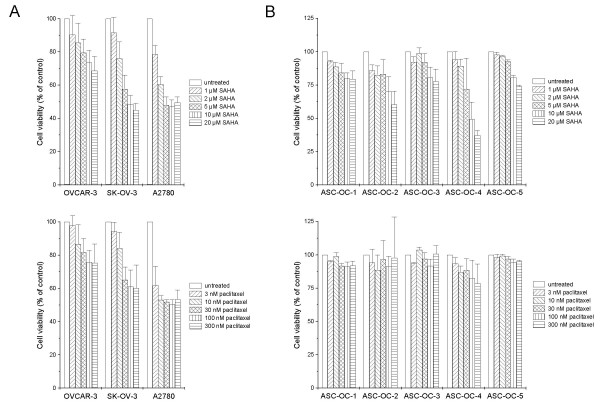
Cytotoxic activity of SAHA and paclitaxel on ovarian cancer cells. **A**, ovarian cancer cell lines; **B**, patient-derived ovarian cancer cells. Cells were incubated with SAHA or paclitaxel for 72 hours, and cytotoxic effects were assessed by Alamar Blue assay. Cell viabilities are expressed as the ratio of absorbance of treated to untreated cells. Means ± SD of 2 (ASC-OC-1), 3 (ASC-OC-2 to ASC-OC-5), or 4 (cell lines) independent experiments in quadruplicates are shown.

Initially, we compared the cytotoxic activities of SAHA and paclitaxel by employing the ovarian carcinoma cell lines OVCAR-3, SK-OV-3, and A2780. SAHA (1–20 μM) and paclitaxel (3–300 nM) were applied in the same concentration range as has been used in other in vitro studies on these agents. Cytotoxicity was monitored by Alamar Blue assay. For all three cell lines, we observed a concentration-dependent cytotoxic effect with increasing doses of the two compounds (Fig. 1A). After treatment with 20 μM SAHA for 72 hours, 45–69% of cells remained viable; after 72 hours of culture with 300 nM paclitaxel, cell viability amounted to 53–75%. Hence, at the concentrations applied, SAHA and paclitaxel induced comparable cytotoxicity in the three cell lines tested.

To compare the potential activities of SAHA and paclitaxel against ovarian carcinoma cells *ex vivo*, we performed cytotoxicity assays with cell isolates from five patients with ovarian cancer. Four of the five ascites samples were obtained from untreated patients, one had been treated with a single dose of carboplatin/paclitaxel (ASC-OC-4). As shown in Fig 1B, SAHA evoked a dose-dependent cytotoxic effect in all five isolates. A 72-h exposure to 20 μM SAHA resulted in 21% (ASC-OC-1) to 63% (ASC-OC-4) cell killing. In contrast, four of the five isolates were resistant to paclitaxel (i.e., <10% cell death at 300 nM paclitaxel for 72 hours). Only isolate ASC-OC-4 was susceptible to this agent, with 21% cell killing observed at 72 hours by 300 nM of paclitaxel. These *ex vivo *observations were in part reflected by the clinical outcome of the chemotherapy: the four patients who had been chemotherapeutically naive at the time of ascites fluid removal were subsequently treated with six cycles of a paclitaxel/carboplatin combination therapy (paclitaxel at a dose of 175 mg/m^2 ^and carboplatin at area under the concentration-time curve of 5 mg/ml/min); in two of them (ASC-OC-1 and -3), no sign of response to treatment was noted.

### Comparison of the caspase-3-activating potencies of SAHA and paclitaxel

**Figure 2:**
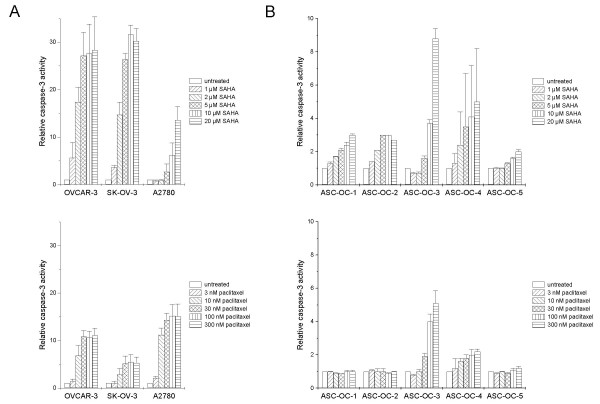
Effect of SAHA and paclitaxel on caspase-3 activity in ovarian cancer cells. **A**, ovarian cancer cell lines; **B**, patient-derived ovarian cancer cells. Cells were incubated with SAHA or paclitaxel for 24 hours, and caspase-3 activity was measured using the fluorogenic substrate Ac-DEVD-AFC. Relative caspase-3 activities are the ratio of treated cells to untreated cells. Means ± SD of 1 (ASC-OC-2 SAHA), 2 (ASC-OC-1, ASC-OC-2 paclitaxel, ASC-OC-4), or 3 (ASC-OC-3, ASC-OC-5, cell lines) independent experiments are shown.

The activation of caspase-3 is a hallmark of apoptotic cell death in many cell types [26]. Accordingly, its activity is considered as an appropriate measure of cytotoxic responsiveness. We assessed whether SAHA and paclitaxel would activate caspase-3 in ovarian cancer cells. First, we examined caspase-3 activities in OVCAR-3, SK-OV-3, and A2780 cells: both SAHA and paclitaxel activated caspase-3 in a concentration-dependent fashion in all three cell lines (Fig. 2A). Second, we analyzed SAHA and paclitaxel for their caspase-3-activating potencies in the patient-derived ovarian cancer cells. As displayed in Fig. 2B, a 24-h treatment with SAHA led to a dose-dependent increase in caspase-3 activity in all five isolates, while a 24-h treatment with paclitaxel had no effect on caspase-3 activity in three of them.

### Assessment of the cytotoxic effects of the combination of SAHA and paclitaxel on ovarian cancer cells

**Figure 3:**
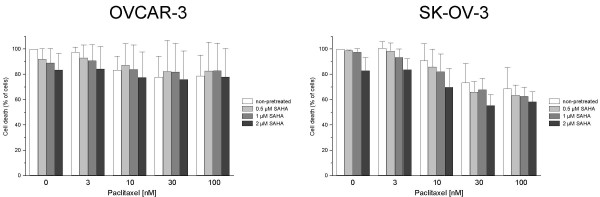
Combination effect of SAHA and paclitaxel on ovarian cancer cells. Four hours after treatment with SAHA, OVCAR-3 and SK-OV-3 cells were exposed to paclitaxel for further 72 hours. Cytotoxic effects were assessed by Alamar Blue assay. Cell viabilities are expressed as the ratio of absorbance of treated to untreated cells. Means ± SD of 3 independent experiments in quadruplicates are shown.

HDIs have been shown to favourably interact with other treatment regimen, such as ionising radiation or chemotherapy [10]. We thus evaluated whether SAHA would cooperate with paclitaxel in exerting cytotoxic effects on ovarian cancer cells. OVCAR-3 and SK-OV-3 cells were pretreated with 0.5, 1, or 2 μM SAHA for 4 hours, and cultured with paclitaxel for further 72 hours. Cytotoxicity was examined by Alamar Blue assay. As presented in Fig. 3, the combined treatment of SAHA and paclitaxel led to an additive effect in SK-OV-3 cells and a less-than-additive effect in OVCAR-3 cells.

### Comparison of SAHA and paclitaxel to induce cell death in drug-resistant ovarian cancer cells

**Figure 4:**
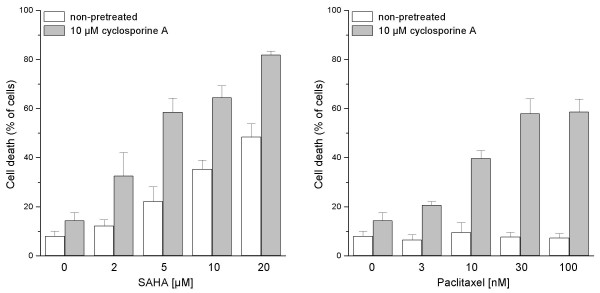
Effect of SAHA and paclitaxel on drug-resistant 2780AD cells. CsA was applied 1 hour before SAHA or paclitaxel, cells were then incubated for 72 hours. Cell death was determined by cytofluorometric analysis of PI uptake. Means ± SD of 3 independent experiments are shown.

Resistance to chemotherapy is frequently associated with the overexpression of ATP-binding cassette drug transporter proteins like Pgp [27]. To investigate the role of Pgp overexpression for the responsiveness of ovarian cancer cells to SAHA and paclitaxel, we employed a drug-resistant variant of the A2780 cell line, the Pgp-overexpressing 2780AD cells [28]. Fig. 4 shows that 2780AD cells were totally resistant to paclitaxel at the doses applied (100 nM paclitaxel for 72 hours). In contrast, a 72-h treatment with SAHA evoked a pronounced cytotoxic effect on these cells, with up to 49% cell killing. In addition, we assessed whether the paclitaxel resistance as well as the SAHA sensitivity could be modulated by the Pgp inhibitor CsA. As demonstrated in Fig. 4, preadministration of 10 μM CsA potently restored the cytotoxic activity of paclitaxel on 2780AD cells, resulting in up to 59% cell death. With respect to SAHA, CsA caused a moderate enhancement of cell killing in 2780AD cells, with a maximum 2.6-fold amplification of SAHA's cytotoxicity being observed at 2 and 5 μM SAHA.

### SAHA treatment leads to histone hyperacetylation in ovarian cancer cells

**Figure 5:**
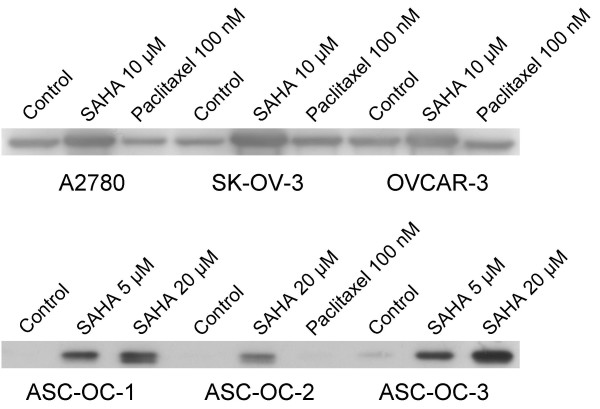
Effect of SAHA and paclitaxel on the acetylation status of histone H3. Cells were treated with SAHA or paclitaxel for 24 hours. Total protein (30 μg) was separated by a 15% polyacrylamid gel. Western blot analyses were performed with antibodies against acetylated histone H3.

The effect of SAHA on histone acetylation was examined in order to confirm that it had effectively inhibited the enzymatic activity of histone deacetylases. Firstly, OVCAR-3, SK-OV-3, and A2780 cells were incubated with 10 μM SAHA or 100 nM paclitaxel for 24 hours, and the acetylation status of histone H3 was analyzed by Western blot using an acetylated H3-specific antibody: treatment with SAHA induced histone H3 hyperacetylation in all three cell lines (Figure 5). Secondly, three of the five ascites samples were exposed to either 5 or 20 μM SAHA (ASC-OC-1 and -3) or to 20 μM SAHA or 100 nM paclitaxel (ASC-OC-2) for 24 hours: SAHA induced a dose-dependent accumulation of acetylated histone H3 in the patient-derived cancer cells. Paclitaxel had no effect on the acetylation status of H3, neither in the cell lines nor in the ascites sample tested.

## Discussion

To the best of our knowledge, this is the first study that systematically compares an HDI and a conventional chemotherapeutic agent for their treatment efficacy in patient-derived cancer cells. HDIs hold promise as a powerful new generation of anticancer drugs. Numerous *in vitro *and animal studies have demonstrated that they can induce differentiation and apoptosis, inhibit cell proliferation, and exert immune stimulatory and antiangiogenic activities [reviewed in [7,8,10,29]]. HDIs have also been shown to enhance the antitumour efficiency of other therapeutic regimens, such as ionizing radiation or chemotherapy. In addition, early-phase clinical trials indicate that HDIs have anticancer activity in a variety of solid and haematological malignancies. However, despite all these studies supporting the development of HDIs for clinical use, no superiority of these agents over conventional antineoplastic agents has yet been directly proven. Here we present evidence that SAHA might be more effective than paclitaxel in the treatment of ovarian cancer.

In order to establish the doses of SAHA and paclitaxel that would induce comparable cell killing in ovarian cancer cells, we initially monitored the cytotoxic activity of these compounds in three ovarian cancer cell lines. SAHA at concentrations of 1 to 20 μM was found to be as efficient in eliciting cell death as paclitaxel at concentrations of 3 to 300 nM. The killing efficiency of the latter was observed to saturate at 100 to 300 nM. This observation is in concordance with findings in clinical studies, in which paclitaxel did not seem to show dose-related efficacy in patients with ovarian cancer [30]. In the patient-derived ovarian cancer cells, SAHA and paclitaxel were of very different effectiveness: while all five isolates responded to SAHA, four of them were unresponsive to paclitaxel. This observation made *ex vivo *had a correlative *in vivo*: two patients were resistant to paclitaxel/carboplatin treatment. However, the number of patient samples used was rather small; thus, this study should not be generalised without additional analyses. The doses of SAHA required to induce cell death in the patient-derived cells were relatively high (5–20 μM); these concentrations would be difficult to achieve in patients without severe toxic side effects [16,18]. Yet, this limitation may be overcome by intraperitoneal administration of SAHA allowing to increase the dose without increasing systemic adverse side effects. An advantage of intraperitoneal chemotherapy in the treatment of ovarian cancer has already been demonstrated in clinical trials [31-33]. Since HDIs have been shown to cooperate with other treatment regimen in exerting antitumour effects [10], we also assessed whether SAHA would enhance the cytotoxic activity of paclitaxel in ovarian cancer cells. Yet, no more than additive effects were seen with this combination treatment. However, this observation is consistent with recent studies, which show that pretreatment with HDIs can enhance the cytotoxicity of DNA-targeting agents such as etoposide, but not that of drugs which do not target the DNA, like the antimetabolite 5-fluorouracil or the microtubule-active vincristine [34,35].

Treatment failure in ovarian cancer therapy is largely due to chemotherapy resistance [3]. One hallmark of chemoresistance is the overexpression of the Pgp drug efflux pump, which confers resistance to a variety of drugs including the taxanes [27]. Here, we have demonstrated that Pgp-overexpressing 2780AD cells are indeed fully resistant to paclitaxel but sensitive to SAHA. This is consistent with previous findings in which SAHA was shown to induce cell death in Pgp-expressing T-cell leukaemia and colon carcinoma cells [36,37]. So, our data on ovarian cancer cells further substantiate that SAHA is capable of killing cancer cells irrespective of their Pgp status. However, although Pgp expression correlates to chemoresistance *in vitro*, its *in vivo *significance in ovarian cancer is unclear [3]. But as histone hypoacetylation is involved in the silencing of a number of chemotherapeutic sensitivity genes, a concerted reexpression of such genes – as accomplished by SAHA as well as other HDIs – holds promise to overcome resistance [38].

The importance of caspases for SAHA- [39] as well as for paclitaxel-induced cell death is a controversial issue. Some reports point to a critical role of caspases for SAHA-mediated cell death [35,40,41], whereas others suggest a caspase-independent mechanism [37,42]. Likewise, paclitaxel-triggered cell death has been reported to proceed in a caspase-dependent [43,44] or -independent [45,46] manner. Here, in using the activation of caspase-3 as a measure of cytotoxic responsiveness, we do not wish to imply that caspase-3 activation would be a necessary prerequisite for SAHA- or paclitaxel-induced cell death. Clearly, the sheer activation of a caspase does not necessarily implicate its requirement for cell death. As a matter of fact, we did not observe a strict correlation between cytotoxic response and caspase-3 activation, especially in the cell lines: for example, while SAHA and paclitaxel induced comparable cytotoxicity in OVCAR-3 and SK-OV-3 cells, SAHA provoked a much higher increase in caspase-3 activity in these cell lines. Nevertheless, the caspase-3 activities observed in the patient-derived cancer cells corroborate the data obtained by Alamar Blue assay: caspase-3 activity was induced by SAHA in all five isolates, but in only two of them by paclitaxel.

## Conclusion

In conclusion, we have shown that SAHA is able to kill paclitaxel-resistant ovarian cancer cells from patients. Since SAHA induced cell death *ex vivo*, it might be a promising agent for ovarian cancer therapy *in vivo*. However, such conclusion should be taken cum grano salis due to the small number of patient samples analysed in our study. Nonetheless, as two of the patients showed no response to paclitaxel/carboplatin treatment, it is tempting to speculate that these patients might have benefited from treatment with SAHA.

## Abbreviations

CsA: Cyclosporine A

HDIs: histone deacetylase inhibitors

Pgp: P-glycoprotein

PI: propidium iodide

SAHA: suberoylanilide hydroxamic acid

## Competing interests

This work was supported by the "Wilhelm Sander-Stiftung, Neustadt/Donau".

## Authors' contributions

JS participated in the study design, interpreted the data, and drafted the manuscript. JG, SP, and CS were responsible for acquisition of data. RO and AB assisted in the interpretation of results and revised the article critically. GL evaluated the patient samples for tumour content and assisted in the analysis and interpretation of results. JFB designed and directed the study and helped to draft the manuscript. All authors read and approved the final manuscript.

## Pre-publication history

The pre-publication history for this paper can be accessed here:
